# Focused ultrasound-mediated blood–brain barrier modulation is associated with adult hippocampal neurogenic responses linked to zinc-dependent signaling

**DOI:** 10.7150/thno.133902

**Published:** 2026-08-10

**Authors:** Bo Young Choi, Jaewoo Shin, Chanho Kong, Min Kyu Park, Dae Ki Hong, Young-soo Chung, Sang Won Suh, Won Seok Chang

**Affiliations:** 1 Department of Physical Education, Hallym University, Chuncheon, 24252, Republic of Korea.; 2 Institute of Sports Science, Hallym University, Chuncheon, 24252, Republic of Korea; 3 Medical Device Development Center, Daegu-Gyeongbuk Medical Innovation Foundation (K-MEDI Hub), Daegu, 41061, Republic of Korea; 4 Department of Neurosurgery, Washington University School of Medicine, Saint Louis, MO, 63110, USA; 5 Department of Physiology, Hallym University, College of Medicine, Chuncheon, 24252, Republic of Korea; 6 Department of Pathology and Laboratory Medicine, Emory University School of Medicine, Atlanta, GA, 30322, USA; 7 Department of Neurosurgery, Brain Research Institute, Yonsei University College of Medicine, Seoul, 03722, Republic of Korea; 8 Department of Biomedical Systems Informatics, Brain Korea 21 PLUS, Yonsei University College of Medicine, Seoul, 03722, Republic of Korea

**Keywords:** focused ultrasound, adult hippocampal neurogenesis, vesicular zinc, zinc transporter 3, blood–brain barrier

## Abstract

**Rationale:**

Transcranial focused ultrasound (FUS)-mediated blood-brain barrier (BBB) modulation is a promising non-invasive therapeutic strategy for targeted brain drug delivery. However, its direct regenerative potential to actively remodel the brain microenvironment and promote adult hippocampal neurogenesis remains largely unexplored owing to elusive molecular mediators. Here, we investigated whether zinc-dependent signaling contributes to adult hippocampal neurogenic responses following FUS-mediated BBB modulation.

**Methods:**

We integrated pharmacological and genetic approaches in adult rodent models subjected to hippocampal-targeted low-intensity FUS with microbubbles. Neural progenitor proliferation and differentiation were quantified using BrdU and DCX labeling. To assess the contributions of extracellular/labile zinc availability and ZnT3-associated vesicular zinc physiology, intracerebroventricular zinc chelation with CaEDTA was performed in rats, and ZnT3^˗/˗^ mice were used. Molecular assays and exploratory bulk RNA sequencing were conducted to characterize candidate downstream molecular pathways.

**Results:**

FUS-mediated BBB modulation significantly increased dentate gyrus progenitor proliferation, neuroblast abundance, and newborn neuron survival. These effects were markedly attenuated by acute zinc chelation and were not observed in ZnT3^˗/˗^ mice, indicating that intact zinc availability and ZnT3-associated zinc physiology are required for the full FUS-associated neurogenic response. FUS was associated with increased expression of brain-derived neurotrophic factor, Zrt-/Irt-like protein 3 (ZIP-3), and Piezo1 proteins, and zinc chelation attenuated these increases. ZIP-3 and Piezo1 signals overlapped with NeuN-positive cells, suggesting neuronal enrichment. In addition, exploratory bulk transcriptomic profiling of whole hippocampal tissue revealed candidate signatures associated with neurovascular and glial responses following FUS in ZnT3^+^/^+^ mice, whereas the corresponding transcriptomic responses appeared reduced or altered in ZnT3^˗/˗^ mice.

**Conclusions:**

Our findings support a framework in which intact zinc availability, including ZnT3-associated zinc physiology, contributes to adult hippocampal neurogenic responses following FUS-mediated BBB modulation. This work provides a mechanistic framework for the development of ultrasound-guided therapeutic strategies aimed at promoting repair-associated plasticity in neurological disorders.

## Introduction

Adult neurogenesis, the generation of new neurons in the mature brain, has emerged as a transformative concept in neuroscience. Once believed to occur only during early development, neurogenesis is now well established to persist throughout adulthood in discrete regions, most notably the subgranular zone (SGZ) of the hippocampal dentate gyrus (DG) and the subventricular zone lining the lateral ventricles 1,2. Newly generated neurons integrate into existing circuits and contribute to key cognitive functions, including learning, memory formation, and cognitive flexibility 3,4. Conversely, reduced neurogenesis is linked to neurodegenerative diseases, mood disorders, and age-related cognitive decline 5,6, underscoring the importance of maintaining neurogenic capacity for normal brain function and resilience against neurological disorders.

Low-intensity transcranial focused ultrasound (FUS) combined with intravenous microbubbles produces a focal, transient, and reversible increase in blood–brain barrier (BBB) permeability, allowing region-specific entry of circulating agents into targeted brain areas 7-10. Microbubble cavitation transiently modulates tight junctions, enhances caveolae-mediated transcytosis, and attenuates efflux transporter activity 11-13. Clinical translation of FUS-mediated BBB modulation has advanced through multiple targeting and monitoring strategies. Magnetic resonance imaging (MRI)-guided systems enable anatomical targeting and post-treatment assessment of BBB permeability, whereas neuronavigation-guided approaches 14 and ultrasound-based cavitation monitoring, including real-time two-dimensional cavitation mapping 15, expand procedural accessibility and acoustic safety feedback. These developments support the feasibility of spatially precise and controllable BBB modulation for neurological disorders 16-18. Preclinical studies further suggest that hippocampus-targeted FUS can influence the local neurovascular microenvironment by engaging inflammatory and glial responses. Under defined acoustic conditions, FUS has been associated with increased progenitor proliferation, enhanced newborn neuron survival, and improved hippocampus-dependent behavior 19-26. These synergistic capabilities suggest therapeutic potential in neurological disorders 27-29. However, the molecular signals linking FUS-mediated BBB modulation to these neurogenic responses remain incompletely defined.

Zinc, an essential trace element enriched in the hippocampus, has received increasing attention as a modulator of synaptic plasticity and neuro-genesis 30,31. A substantial pool of brain zinc is stored in presynaptic vesicles of hippocampal mossy fiber terminals through the activity of zinc transporter 3 (ZnT3), and synaptically released zinc can influence signaling pathways involving neurotrophins, ion channels, and growth factor receptors 32-34. Previous studies have shown that reduced zinc availability, extracellular zinc chelation, or ZnT3 deficiency impairs adult hippocampal progenitor proliferation, neuroblast production, and neuronal differentiation 35-39. Together, these findings support zinc availability, including ZnT3-associated vesicular zinc physiology, as an important permissive determinant of adult hippocampal neurogenesis.

Given these observations, we hypothesized that FUS-mediated BBB modulation may engage zinc-dependent signaling within the adult hippocampal neurogenic niche. Rather than assuming a single zinc source or a cell-autonomous pathway, we asked whether extracellular/labile zinc availability and ZnT3-associated zinc physiology contribute to the neurogenic responses observed after FUS. We also asked whether FUS was associated with changes in candidate zinc-related molecular responses, including brain-derived neurotrophic factor (BDNF), Zrt-/Irt-like protein 3 (ZIP-3), and the mechanosensitive ion channel Piezo1, and whether exploratory transcriptomic profiling would identify broader niche-level signatures associated with neurovascular and glial responses.

In this study, we combined hippocampal-targeted FUS in adult rodents with histological analyses, pharmacological zinc chelation, and genetically modified ZnT3 knockout (KO) models to test whether brain zinc contributes to adult hippocampal neurogenic responses following FUS-mediated BBB modulation. We quantified progenitor proliferation, neuroblast abundance, and the survival and differentiation of newborn cells and further assessed associated changes in BDNF, ZIP-3, and Piezo1 protein expression together with exploratory bulk RNA sequencing to identify candidate early transcriptional signatures related to FUS exposure and ZnT3 status.

## Materials and Methods

### Animals and ethics statement

Sprague–Dawley male rats (2–3 months old, 250–350 g) were obtained from DBL (Chungcheongbuk-do, Republic of Korea). ZnT3^+^/^+^ (WT) and ZnT3^˗/˗^ (KO) male mice (3 months old, 25–30 g; C57BL/6 × Sv129 hybrid background) were provided by Dr. Jae-Young Koh (Department of Neurology, University of Ulsan College of Medicine, Republic of Korea). In this study, only male animals were included to minimize sex-related variability in the multi-arm experimental design, thereby precluding assessment of sex-dependent effects. Animals were housed in groups of three per cage under controlled environmental conditions (22 ± 2 °C, 55 ± 5% relative humidity, and a 12-h light/dark cycle; lights on at 07:00) and had ad libitum access to standard laboratory chow (Purina, Gyeonggi-do, Republic of Korea) and water. To minimize stress associated with transportation and adaptation to the housing environment, animals were acclimated for 1 week before experimental procedures. All animal procedures were approved by the Institutional Animal Care and Use Committee of Yonsei University (IACUC No. 2016-0339) and were conducted in accordance with the National Institutes of Health Guide for the Care and Use of Laboratory Animals. The study is reported in accordance with the ARRIVE (Animal Research: Reporting of *In Vivo* Experiments) guidelines 40.

### FUS procedure

**Figure 1:**
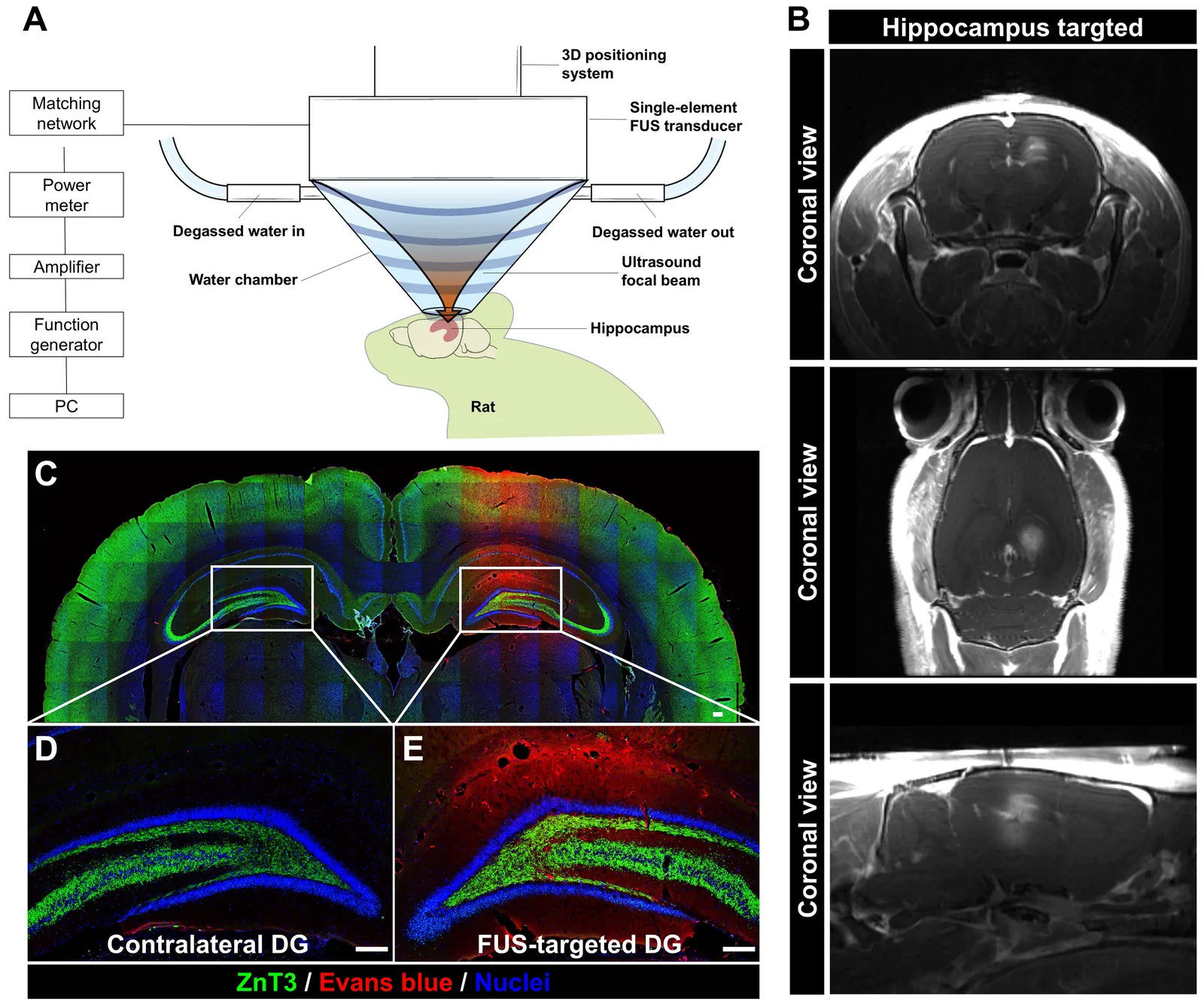
** Experimental setup and post-sonication assessment of a localized increase in BBB permeability after FUS targeted to the hippocampus. (A)** Schematic of stereotactically targeted FUS delivery to the right hippocampus using a 0.5-MHz single-element transducer, intravenous microbubble administration, a degassed water-filled acoustic coupling cone, and stereotaxic positioning. **(B)** Gadolinium-enhanced T1-weighted MRI acquired immediately after sonication showing focal contrast enhancement at the FUS-targeted right hippocampus, consistent with increased BBB permeability. **(C)** Representative merged fluorescence image of a coronal brain section showing Evans blue fluorescence (red), ZnT3 immunoreactivity (green), and nuclei counterstained with DAPI (blue). **(D, E)** Corresponding higher-magnification images of the matched contralateral DG **(D)** and the FUS-targeted ipsilateral DG **(E)**. Evans blue fluorescence was observed in the FUS-targeted ipsilateral DG, whereas little or no signal was detected in the matched contralateral DG. Scale bars = 200 μm.

Rats and mice were anesthetized with an intraperitoneal injection of ketamine (75 mg/kg) and xylazine (4 mg/kg), positioned in a stereotaxic frame, and placed on a thermostatically controlled heating pad. Core body temperature was monitored using a rectal probe and maintained at approximately 37 °C. Respiratory pattern and depth of anesthesia were monitored throughout the procedure. Animals remained under anesthesia throughout the FUS procedure and post-sonication MRI confirmation. The sonication duration was 120 s, and the total procedure time from anesthesia induction to completion of MRI confirmation was approximately 20 min. A single-element spherically FUS transducer (center frequency: 0.5 MHz; diameter: 51.7 mm; radius of curvature: 63.2 mm; model H-107MR, Sonic Concepts, Bothell, WA, USA) was used to target the right dorsal hippocampus. The transducer was driven by a function generator (Agilent, 33220A) connected to a 50 dB RF power amplifier (ENI 240L) and monitored via a power meter (Agilent, E4419B). An impedance matching network (Sonic Concepts) was used to optimize transmission efficiency. For acoustic coupling, a water-filled cone (degassed, distilled water) sealed with a thin polyurethane membrane was attached to the transducer (Figure 1A). Ultrasound gel was applied between the membrane and the exposed skull surface to facilitate efficient coupling. The FUS focal point was stereotaxically aligned with the dorsal hippocampus using the following coordinates: ~3.0 mm lateral to midline, ~3.8 mm posterior to bregma, and an appropriate depth to target the DG. A lipid-shelled microbubble contrast agent (Definity®, Lantheus Medical Imaging; 1.1–3.3 μm diameter) was diluted in sterile saline and injected intravenously via the tail vein (0.02 mL/kg) approximately 10 s before sonication. FUS was delivered using 10-ms bursts at a 1 Hz pulse repetition frequency for a total of 120 s. The free-field focal pressure was measured under the same transducer configuration and adjusted for estimated skull attenuation under the present experimental conditions 9,41. Therefore, the reported peak-negative pressure of 0.25–0.30 MPa represents the estimated in situ focal pressure at the hippocampal target 42. To verify successful BBB modulation, T1-weighted MRI was performed immediately after sonication using a 9.4 T scanner (Bruker Biospec 94/20). Gadobutrol (0.2 mL/kg; Bayer Schering) was administered intravenously, and pre- and post-contrast images were acquired to assess signal enhancement in the targeted hemisphere 43. MRI acquisition parameters were as follows: repetition time ≈ 350 ms, echo time = 5.4 ms, flip angle = 40°, matrix = 256 × 256, field of view = 35 mm, and two signal averages.

### Experimental design

To investigate the contribution of zinc-dependent signaling to adult hippocampal neurogenic responses associated with FUS-mediated BBB modulation, we conducted three complementary experiments. Across the three experiments, animals assigned to FUS treatment underwent low-intensity FUS targeted to the right hippocampus using predefined acoustic parameters described above, with concurrent microbubble administration. Animals assigned to BrdU-based neurogenesis analyses received BrdU (50 mg/kg, intraperitoneally) twice daily for four consecutive days, beginning 24 h after FUS or the corresponding sham procedure, to label dividing cells and permit subsequent assessment of the survival and lineage differentiation of BrdU-labeled cells.

Experiment 1—Characterization of BBB per-meability, zinc-related responses, and hippocampal neurogenesis following FUS-mediated BBB modulation: To characterize BBB permeability, zinc-related responses, and the temporal profile of hippocampal neurogenesis following unilateral FUS-mediated BBB modulation, rats underwent FUS targeted to the right hippocampus. The contralateral hippocampus served as a within-animal comparator for paired assessment of ipsilateral changes. BBB permeability was qualitatively assessed by Evans Blue fluorescence. Evans Blue dye (2% in sterile saline, 100 mg/kg; Sigma-Aldrich, St. Louis, MO, USA) was administered intravenously after FUS. Separate cohorts were euthanized 4 or 24 h after FUS to assess BBB permeability and TSQ-detectable zinc signal by Evans Blue extravasation and TSQ histofluorescence, respectively. On day 5 after FUS, ZnT3 immunoreactivity, progenitor cell proliferation, and neuroblast abundance were assessed, whereas on day 21, the survival and neuronal and astroglial differentiation of BrdU-labeled cells were evaluated.

Experiment 2—Effect of extracellular zinc chelation on hippocampal neurogenic responses following FUS-mediated BBB modulation: To determine whether labile extracellular zinc availability contributes to the FUS-associated neurogenic response, a separate cohort of rats was assigned to sham, vehicle-treated FUS, CaEDTA-treated FUS, or ZnEDTA-treated FUS groups. Immediately after FUS, animals in the CaEDTA-treated FUS group received an intracerebroventricular infusion of CaEDTA (100 mM, 5 µL; 500 nmol) into the right lateral ventricle over approximately 5 min using a Hamilton microsyringe. Animals in the vehicle-treated FUS and ZnEDTA-treated FUS groups received equal-volume intracerebroventricular infusions of saline or ZnEDTA (100 mM, 5 µL), respectively, using the same procedure. Saline served as the vehicle control, whereas ZnEDTA served as a zinc-saturated EDTA control for nonspecific effects associated with administration of the chelator complex.

Sham-operated animals underwent the corresponding anesthesia and surgical procedures but received neither microbubbles nor FUS. For histological analyses, animals were euthanized on day 5 or 21 after FUS or the corresponding sham procedure. At the 5-day endpoint, progenitor cell proliferation and neuroblast abundance were assessed, whereas the 21-day endpoint was used to assess the survival and neuronal and astroglial differentiation of BrdU-labeled cells. In addition, a separate set of animals from each Experiment 2 group was euthanized 24 h after FUS or the corresponding sham procedure for Western blot analysis of early molecular responses.

Experiment 3—Contribution of ZnT3-associated zinc signaling to hippocampal neurogenic responses following FUS-mediated BBB modulation: To assess whether ZnT3-associated vesicular zinc physiology contributes to the full FUS-associated neurogenic response, WT and ZnT3^˗/˗^ mice were examined in two separate cohorts with distinct comparator structures. Experiment 3A—ZnT3 histological cohort: WT and ZnT3^˗/˗^ mice underwent unilateral FUS targeted to the right hippocampus and received BrdU as described above. The contralateral hippocampus served as a matched within-animal comparator for assessment of ipsilateral changes within each genotype. Animals were euthanized on day 5 after FUS, and BrdU+, DCX+, and BrdU+/DCX+ cells in the DG were quantified. Experiment 3B—Exploratory ZnT3 transcriptomic cohort: A separate cohort was assigned to four independent groups: WT-sham, WT-FUS, ZnT3^˗/˗^-sham, and ZnT3^˗/˗^-FUS. Fresh hippocampal tissue was collected 24 h after sham or FUS treatment for exploratory bulk RNA sequencing to identify candidate early transcriptional signatures associated with FUS exposure and ZnT3 genotype. The two cohorts were analyzed independently.

Animals were randomly assigned to experimental groups before the FUS or sham procedure using a computer-generated random-number sequence. Outcome quantification and image analysis were performed by an investigator blinded to group allocation.

### Tissue preparation

Rats and mice were deeply anesthetized with urethane (1.5 g/kg, intraperitoneally) and transcardially perfused with ice-cold phosphate-buffered saline (PBS), followed by 4% paraformaldehyde (PFA) in PBS. Brains were extracted and post-fixed in 4% PFA for 1 h at 4 °C, then transferred to 30% sucrose in PBS at 4 °C for cryoprotection until completely submerged. After cryoprotection, brains were rapidly frozen and coronally sectioned at 30 μm using a cryostat microtome (CM1850; Leica, Wetzlar, Germany). Sections were collected and stored in cryoprotectant solution until further processing for histological or immunofluorescence analyses.

### Fluorescence zinc staining

N-(6-methoxy-8-quinolyl)-para-toluenesulfona-mide (TSQ)-detectable histochemically reactive zinc in the hippocampal mossy fiber region was visualized using the fluorescent zinc indicator TSQ (Molecular Probes, Eugene, OR, USA). For TSQ staining, a separate subset of animals was processed without perfusion fixation to preserve histochemically reactive zinc. Fresh, unfixed brain tissue was obtained by rapidly freezing extracted brains in powdered dry ice without prior perfusion fixation, following established methods 44. Coronal cryosections containing the dorsal hippocampus were air-dried and incubated for 1 min in TSQ staining solution (4.5 μM TSQ prepared in 140 mM sodium barbital and 140 mM sodium acetate buffer, pH 10.5). Sections were then rinsed for 1 min in 0.9% saline to remove unbound dye. TSQ selectively binds free or loosely bound Zn²⁺, producing blue fluorescence (excitation ~360 nm; emission ~490 nm) in zinc-rich regions, including the DG and mossy fiber projections. Stained sections were imaged immediately using an Olympus upright fluorescence microscope equipped with a UV excitation filter. Fluorescent signals were captured with a cooled CCD camera (Hamamatsu Photonics, Bridgewater, NJ, USA) and analyzed using Infinity 3 software (Lumenera, Ottawa, Canada). For quantitative comparisons, TSQ fluorescence intensity was measured using the ImageJ software with identical exposure conditions applied across all samples. Five anatomically matched coronal sections containing the dorsal hippocampus were analyzed per rat. For each section, TSQ fluorescence intensity was measured separately in the FUS-treated ipsilateral and matched contralateral hippocampi. The measurements from the five sections were averaged separately for each hemisphere to generate a single animal-level value per hemisphere.

### Immunohistochemistry

To visualize proliferating cells and immature neurons, chromogenic immunohistochemistry was performed using diaminobenzidine (DAB) as the detection substrate. Fixed free-floating sections were first incubated in 1.2% hydrogen peroxide in PBS for 20 min to eliminate endogenous peroxidase activity, followed by PBS washes. For BrdU detection, sections were pretreated with 2 N HCl to denature DNA, neutralized with 0.1 M borate buffer, and washed in PBS before blocking. Sections were then transferred to a blocking and permeabilization solution containing 0.3% Triton X-100 and 5% normal serum to reduce nonspecific binding and improve antibody penetration. Primary antibody incubation was performed overnight at 4 °C using rat anti-BrdU antibody (1:400; Abcam, Cambridge, UK) to label proliferating cells and guinea pig anti-DCX antibody (1:1000; Millipore, Billerica, MA, USA) to detect immature neurons in the DG. After additional PBS washes, sections were incubated for 2 h at room temperature with biotinylated secondary antibodies (1:250; Vector Laboratories, Burlingame, CA, USA), using biotinylated anti-rat IgG for BrdU and biotinylated anti-guinea pig IgG for DCX detection. Signal amplification was performed using an avidin–biotin–peroxidase complex (ABC kit, Vector Laboratories) for 2 h. DAB development was performed in PBS containing 0.015% hydrogen peroxide until a distinct brown precipitate became visible, with reaction times kept constant across samples. Sections were then mounted onto gelatin-coated slides, air-dried, dehydrated through graded ethanol, cleared in xylene, and coverslipped with permanent mounting medium. Bright-field images were acquired using an Olympus IX70 microscope with consistent illumination and camera settings across all samples.

### Immunofluorescence analysis

Immunofluorescence staining was performed to visualize specific cellular and molecular markers in hippocampal sections. Free-floating sections were first rinsed in PBS and then incubated in a blocking and permeabilization solution containing 0.3% Triton X-100 and 5% normal serum to reduce background staining and improve antibody penetration. Primary antibodies were applied overnight at 4 °C and included rabbit anti-ZnT3 (1:200, Synaptic Systems, Goettingen, Germany) to label synaptic vesicular zinc transporters; rat anti-BrdU (1:400, Abcam) to identify proliferating cells; guinea pig anti-DCX (1:1000, Millipore) to detect immature neurons; rabbit anti-NeuN (1:500, Millipore) for mature neurons; and mouse anti-GFAP (1:200, Millipore) for astrocytes. For double-labeling experiments, primary antibodies generated in different species were co-incubated to enable simultaneous multi-target detection within the same section. Markers such as ZIP-3 (1:500; Alomone Labs, Jerusalem, Israel) and Piezo1 (1:1000; Cell Signaling Technology, Danvers, MA, USA), when examined, were processed using the same staining protocol with correspondingly matched antibodies. After PBS washes, sections were incubated for 2 h at room temperature with fluorophore-conjugated secondary antibodies (1:250; Thermo Fisher Scientific, Waltham, MA, USA) matched to the species of each primary antibody. Fluorophores were selected to minimize spectral overlap and allow clean signal separation during imaging. Nuclear counterstaining was performed using DAPI (1:1000; Thermo Fisher Scientific). Sections were then mounted on gelatin-coated slides and coverslipped using DPX mounting medium (Sigma-Aldrich, Burlington, MA, USA).

### Western blot

To assess protein expression changes in the hippocampus, freshly dissected tissues were homogenized in ice-cold RIPA buffer containing 10 mM Tris-HCl (pH 7.4), 150 mM NaCl, 1% Nonidet P-40, 0.5% sodium deoxycholate, and 0.1% SDS. Homogenates were centrifuged at 14,000 × *g* for 20 min at 4 °C, and the resulting supernatants were collected as total protein lysates and stored at -80 °C until analysis. Protein concentrations were quantified using the Bradford assay to ensure equal loading across samples. For electrophoresis, equal amounts of denatured protein were loaded onto 8% sodium dodecyl sulfate–polyacrylamide gels and separated by molecular weight. Proteins were transferred onto polyvinylidene difluoride membranes and blocked for 2 h at room temperature in 10% skim milk prepared in TBST (50 mM Tris-HCl, pH 7.5; 150 mM NaCl; 0.1% Tween-20) to prevent nonspecific binding. Membranes were incubated overnight at 4 °C with primary antibodies targeting BDNF (1:1000; Abcam), ZIP-3 (1:2000; Alomone Labs), and Piezo1 (1:1000; Cell Signaling Technology). After washing in TBST, membranes were incubated with the appropriate HRP-conjugated secondary antibodies (anti-rabbit or anti-mouse IgG, diluted 1:1000) for 1 h at room temperature. The proteins were visualized using an enhanced chemiluminescence solution (SuperSignal West Pico PLUS, Thermo Fisher Scientific), and blots were analyzed using a LAS 4000 mini (GE Healthcare Life Sciences). The intensity of each band was digitized and analyzed using ImageJ software (NIH) under identical exposure settings to ensure consistent comparisons across experimental groups.

### RNA extraction and sequencing

Fresh hippocampal tissue was collected 24 h after the sham or FUS procedure from 14 mice (WT-sham, n = 4; WT-FUS, n = 3; ZnT3^˗/˗^-sham, n = 4; ZnT3^˗/˗^-FUS, n = 3) for exploratory bulk RNA sequencing to identify candidate early transcriptional signatures associated with FUS exposure and ZnT3 status. Total RNA was extracted using a QIAGEN miRNeasy Mini Kit (Qiagen, Hilden, Germany). A total of 116 ng RNA per sample was used to prepare sequencing libraries using Illumina Stranded Total RNA Prep Ligation with Ribo-Zero Plus following the manufacturer’s protocol. Libraries were sequenced on an Illumina HiSeq 3000 at the Yonsei Genome Center (Seoul, Republic of Korea) to generate paired-end reads.

### Bioinformatic analysis

Raw sequencing data were quality-checked using FastQC, and adapter sequences were trimmed where necessary using Trimmomatic (v0.32). Clean reads were aligned to the mouse reference genome (mm10) using the splice-aware aligner HISAT2 (v2.2.1). Differentially expressed genes were identified using the PyDESeq2 package (v0.4.10). Genes meeting the thresholds of FDR < 0.05 and |log2 FC| > 0.5 were included in downstream pathway analyses. Gene Ontology enrichment was performed using GOATOOLS (v1.4.12), and gene set enrichment analysis was conducted using gseapy (v1.1.3). Data visualization, including heatmap and volcano plot generation, was performed using Python libraries such as matplotlib and seaborn. Illustrations in Figure 7 were created using BioRender.

### Statistical analysis

All data were presented as mean ± standard error of the mean (SEM). Statistical significance was defined at *P* < 0.05 for all analyses. Normality was assessed using the Shapiro–Wilk test, and homogeneity of variance was assessed using the Brown–Forsythe test where applicable. In Experiment 1, comparisons between the FUS-targeted ipsilateral hippocampus and the matched contralateral hippocampus were performed using paired Student’s t-tests after confirming normality of the paired differences. In Experiment 2, four-group comparisons were analyzed using one-way ANOVA followed by Bonferroni-corrected post hoc comparisons when both normality and homogeneity assumptions were satisfied; datasets that satisfied normality but not homogeneity of variance were analyzed using Welch’s ANOVA followed by Games–Howell post hoc comparisons. In Experiment 3, in the ZnT3 histological dataset, genotype-dependent hemispheric differences were analyzed using a two-way mixed ANOVA, with genotype (WT vs ZnT3^˗/˗^) as the between-subject factor and hemisphere (FUS-targeted ipsilateral vs matched contralateral) as the within-subject factor, followed by Bonferroni-corrected post hoc comparisons. The exploratory bulk RNA-seq dataset was obtained from a separate cohort comprising four independent groups (WT-sham, WT-FUS, ZnT3^˗/˗^ -sham, and ZnT3^˗/˗^ -FUS) and was analyzed independently of the histological dataset. All statistical analyses were performed using SPSS (Version 20, IBM SPSS Statistics, Chicago, IL, USA) and GraphPad Prism 10 software (GraphPad Software Inc., San Diego, USA).

## Results

### Localized BBB modulation after unilateral FUS is accompanied by increased hippocampal zinc-associated signals

FUS was stereotactically delivered to the right hippocampus using the experimental configuration shown in Figure 1A. Gadolinium-enhanced T1-weighted MRI obtained immediately after sonication showed focal contrast enhancement in the FUS-targeted right hippocampus (Figure 1B). In addition, coronal sections examined after systemic administration of Evans Blue dye, which binds to serum albumin, showed evident fluorescence in the FUS-targeted ipsilateral DG, whereas little or no signal was detected in the matched contralateral DG (Figure 1C-E). These findings indicate that unilateral FUS targeting of the hippocampus produced a localized increase in BBB permeability.

**Figure 2:**
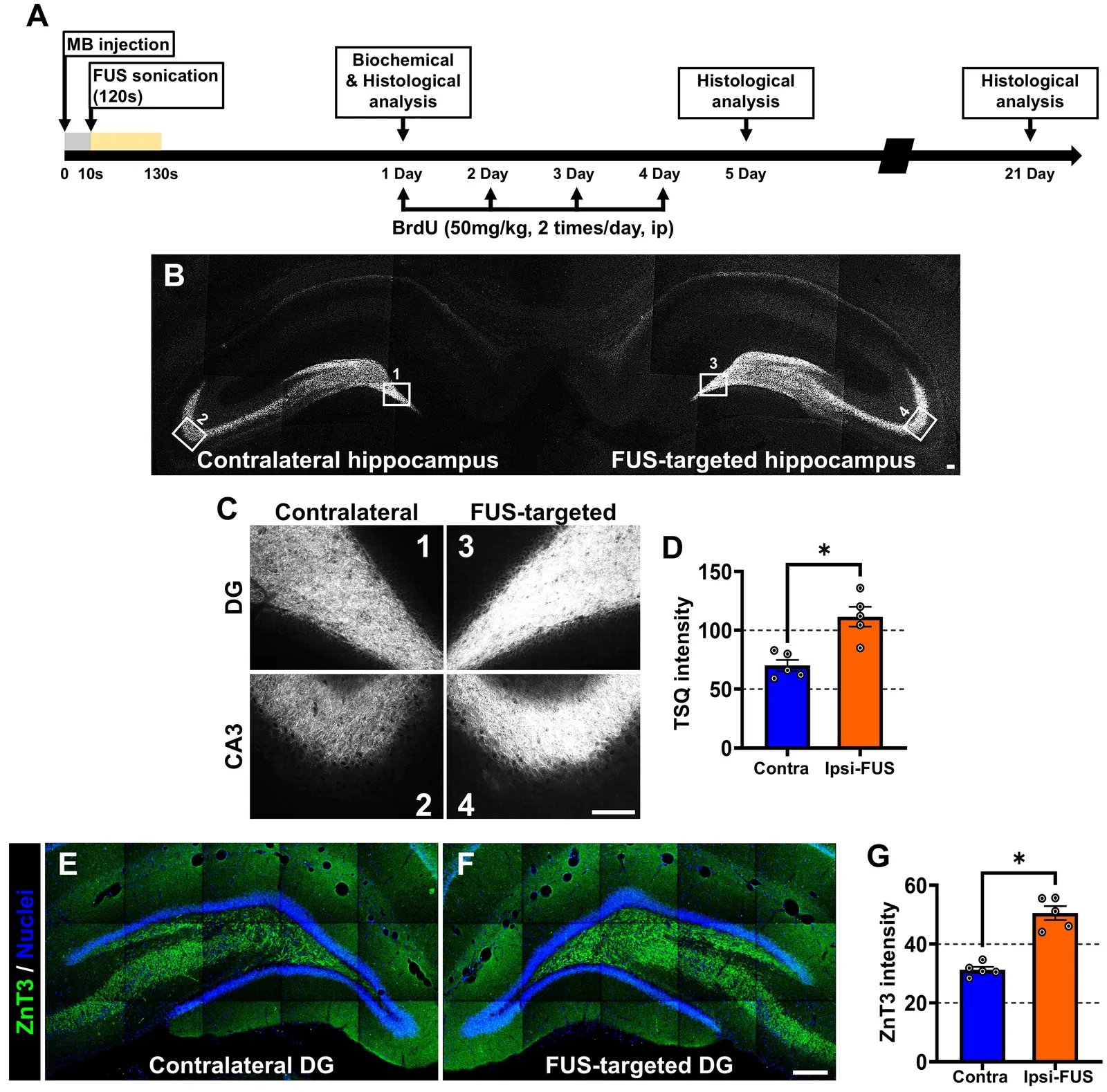
** TSQ histofluorescence and ZnT3 immunoreactivity in the hippocampus after unilateral FUS**. **(A)** Schematic of the experimental timeline. **(B)** Representative low-magnification images of TSQ histofluorescence in the matched contralateral and FUS-targeted ipsilateral hippocampi 24 h after FUS. TSQ fluorescence was detected predominantly along the mossy fiber pathway of the DG and CA3. Scale bar = 100 μm.** (C)** Corresponding higher-magnification images of the mossy fiber pathway in the DG and CA3. Scale bar = 50 μm.** (D)** Quantification of TSQ fluorescence intensity in the mossy fiber pathway of the matched contralateral and FUS-targeted ipsilateral hippocampi. **(E, F)** Representative immunofluorescence images of ZnT3 (green) and DAPI (blue) in the matched contralateral DG **(E)** and FUS-targeted ipsilateral DG **(F)** on day 5 after FUS. Scale bar = 100 μm.** (G)** Quantification of ZnT3 immunofluorescence intensity in the matched contralateral and FUS-targeted ipsilateral DG. Data are presented as mean ± SEM (n = 5 rats for each analysis). For each analysis, matched contralateral and FUS-targeted ipsilateral measurements from the same animals were compared using paired Student’s t-tests. *P < 0.05 versus the matched contralateral hippocampus.

We next examined whether this response was accompanied by changes in hippocampal zinc-associated signals. As shown in the experimental timeline in Figure 2A, TSQ histofluorescence was evaluated 24 h after unilateral FUS. TSQ fluorescence was detected predominantly along the mossy fiber pathway of the DG and CA3 in both hemispheres, and the signal was greater in the FUS-targeted ipsilateral hippocampus than in the matched contralateral hippocampus (Figure 2B-C). Quantitative analysis confirmed a significant increase in TSQ fluorescence intensity in the mossy fiber pathway of the FUS-targeted ipsilateral hippocampus (Figure 2D). ZnT3 immunoreactivity was then examined on day 5 after FUS. Representative images showed stronger ZnT3 immunofluorescence in the FUS-targeted ipsilateral DG than in the matched contralateral DG (Figure 2E-F), and this hemisphere difference was significant on quantitative analysis (Figure 2G). Together, these findings show that unilateral FUS targeted to the hippocampus was associated with a localized increase in BBB permeability, as well as increased TSQ-detectable zinc fluorescence and ZnT3 immunoreactivity in the hippocampus.

### Unilateral FUS is associated with increased proliferation and altered phenotypic distribution of BrdU-labeled cells in the DG

**Figure 3:**
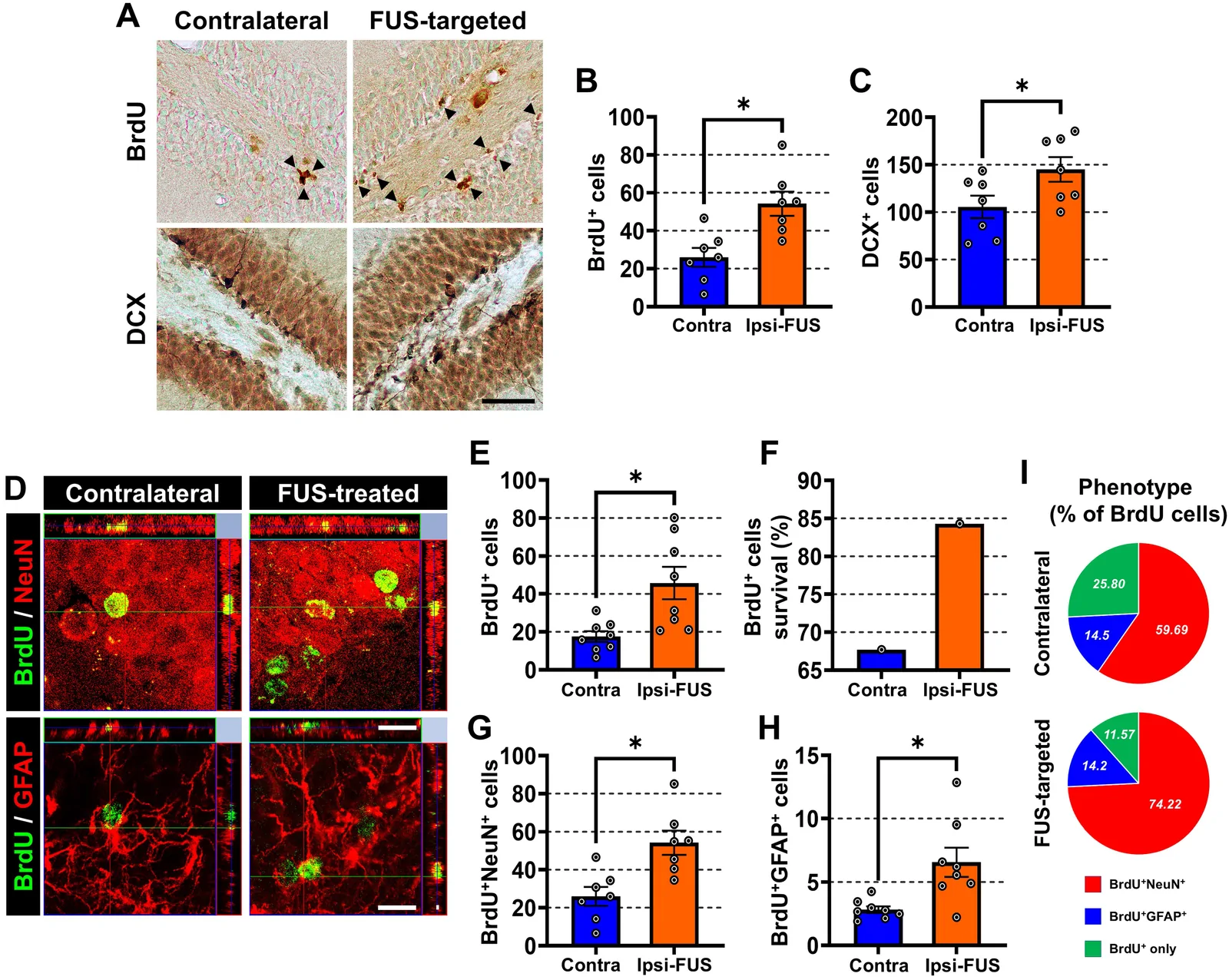
**Proliferation, survival, and phenotypic analysis of BrdU-labeled cells in the DG after unilateral FUS. (A)** Representative immunofluorescence images of the SGZ of the DG in the matched contralateral and FUS-targeted ipsilateral hippocampi on day 5 after FUS, stained for BrdU and DCX. Scale bar = 50 μm. **(B, C)** Quantification of BrdU-positive cells **(B)** and DCX-positive cells **(C)** in the SGZ/GCL of the matched contralateral and FUS-targeted ipsilateral hippocampi on day 5 after FUS.** (D)** Representative higher-magnification immunofluorescence images of the DG on day 21 after FUS, showing BrdU (green) co-labeled with NeuN (red) or GFAP (red). Scale bar = 10 μm. **(E, F)** Quantification of the total number of BrdU-positive cells in the SGZ/GCL **(E)** and the percentage of BrdU-labeled cells surviving at 21 days **(F)** in the matched contralateral and FUS-targeted ipsilateral hippocampi. **(G, H)** Quantification of BrdU/NeuN double-positive cells **(G)** and BrdU/GFAP double-positive cells **(H)** in the DG on day 21 after FUS. **(I)** Percentage of BrdU-labeled cells co-labeled with NeuN or GFAP in the matched contralateral and FUS-targeted ipsilateral hippocampi. Data are presented as mean ± SEM (n = 7 rats for the 5-day analysis in B and C; n = 8 rats for the 21-day analysis in E–I). For each analysis, matched contralateral and FUS-targeted ipsilateral measurements from the same animals were compared using paired Student’s t-tests. *P < 0.05 versus the matched contralateral hippocampus.

Having found that unilateral FUS-mediated BBB modulation was accompanied by increased hippocampal zinc-associated signals, we next examined whether these changes were accompanied by alterations in adult neurogenesis in the DG. Using the experimental timeline shown in Figure 2A, BrdU was administered after FUS to label newly dividing cells, and hippocampal tissue was analyzed 5 days later to assess early proliferative responses. Representative immunofluorescence images showed BrdU-positive and DCX-positive cells along the subgranular zone/granule cell layer (SGZ/GCL), with greater labeling in the FUS-targeted ipsilateral hippocampus than in the matched contralateral hippocampus (Figure 3A). Quantitative analysis confirmed significant increases in both BrdU-positive cells and DCX-positive cells in the SGZ/GCL of the FUS-targeted ipsilateral hippocampus on day 5 after FUS (Figure 3B-C).

We next evaluated the persistence and phenotypic fate of BrdU-labeled cells on day 21 after FUS. Representative images showed BrdU-labeled cells co-localized with NeuN or GFAP in the DG (Figure 3D). The total number of BrdU-positive cells in the SGZ/GCL remained significantly higher in the FUS-targeted ipsilateral hippocampus than in the matched contralateral hippocampus (Figure 3E). In addition, group-level estimates of the percentage of BrdU-labeled cells surviving to 21 days were higher in the FUS-targeted ipsilateral hippocampus (Figure 3F). The numbers of both BrdU/NeuN double-positive cells and BrdU/GFAP double-positive cells were also higher in the FUS-targeted ipsilateral DG (Figure 3G-H). When expressed as proportions of the BrdU-labeled population, the NeuN-positive fraction was increased in the FUS-targeted ipsilateral hippo-campus, whereas the GFAP-positive fraction was similar between hemispheres (Figure 3I). Together, these findings indicate that unilateral FUS was associated with increased early proliferative and immature neuronal markers in the DG and, at the later time point, with an expanded BrdU-labeled cell population together with a phenotypic distribution shifted toward NeuN-positive cells.

### Extracellular zinc chelation attenuates FUS-associated neurogenic responses in the DG

**Figure 4:**
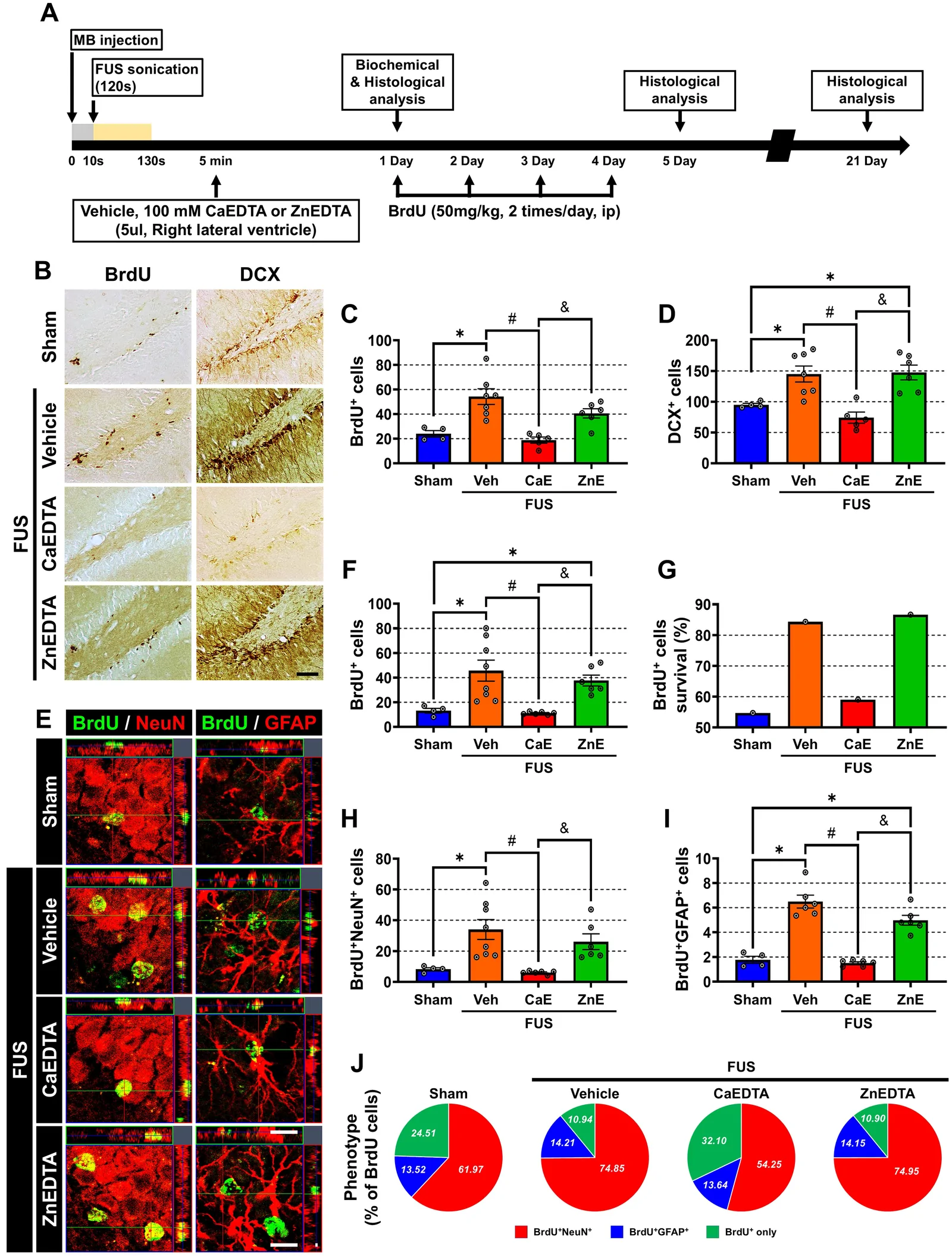
** Effects of vehicle, CaEDTA, and ZnEDTA treatment on FUS-associated neurogenic responses in the DG. (A)** Schematic of the experimental timeline and treatment groups.** (B)** Representative immunofluorescence images of the DG in the sham-operated, vehicle-treated FUS, CaEDTA-treated FUS, and ZnEDTA-treated FUS groups on day 5 after the sham or FUS procedure, stained for BrdU and DCX. Scale bar = 50 μm. **(C, D)** Quantification of BrdU-positive cells** (C)** and DCX-positive cells** (D)** in the SGZ/GCL on day 5 after the sham or FUS procedure across the four groups.** (E)** Representative immunofluorescence images of the DG in the four groups on day 21 after the sham or FUS procedure, showing BrdU and NeuN co-labeling. Scale bar = 20 μm. **(F, G)** Quantification of the total number of BrdU-positive cells in the SGZ/GCL** (F)** and the percentage of BrdU-labeled cells surviving at 21 days** (G)**.** (H, I)** Quantification of BrdU/NeuN double-positive cells** (H)** and BrdU/GFAP double-positive cells** (I)** in the DG on day 21 after the sham or FUS procedure.** (J)** Pie charts summarizing the percentages of BrdU-labeled cells co-labeled with NeuN or GFAP in each group. Data are presented as mean ± SEM (n = 4–7 rats per group for C and D; n = 4–8 rats per group for F–I). Panels C and I were analyzed using one-way ANOVA followed by Bonferroni-corrected post hoc comparisons. Panels D, F, and H were analyzed using Welch’s ANOVA followed by Games–Howell post hoc comparisons. *P < 0.05 versus the sham-operated group, #P < 0.05 versus the vehicle-treated FUS group, and &P < 0.05 versus the CaEDTA-treated FUS group.

To test whether extracellular zinc availability contributes to the FUS-associated neurogenic response, vehicle, CaEDTA, or ZnEDTA was administered into the right lateral ventricle 5 min after sonication, as shown in Figure 4A. On day 5 after FUS, representative images showed increased BrdU and DCX labeling in the vehicle-treated FUS group relative to the sham-operated group, whereas these increases were attenuated in the CaEDTA-treated FUS group; the ZnEDTA-treated FUS group showed a pattern similar to that of the vehicle-treated FUS group (Figure 4B). Quantitative analysis confirmed that the numbers of BrdU-positive and DCX-positive cells in the SGZ/GCL were significantly higher in the vehicle-treated FUS group than in the sham-operated group and were significantly reduced by CaEDTA treatment (Figure 4C-D). In contrast, ZnEDTA did not suppress these FUS-associated increases.

We next examined BrdU-labeled cells on day 21 after FUS to assess longer-term persistence and phenotypic distribution. The total number of BrdU-positive cells in the SGZ/GCL remained higher in the vehicle-treated FUS group than in the sham-operated group and was significantly reduced in the CaEDTA-treated FUS group, whereas the ZnEDTA-treated FUS group remained similar to the vehicle-treated FUS group (Figure 4E-F). Group-level estimates of BrdU-cell survival showed the same overall pattern (Figure 4G). Quantification further showed that the number of BrdU/NeuN double-positive cells was increased in the vehicle-treated FUS group and reduced by CaEDTA treatment (Figure 4H). The number of BrdU/GFAP double-positive cells showed a similar directional change at the level of absolute cell counts (Figure 4I). Consistent with these findings, the phenotype distributions shown in Figure 4J indicated a smaller NeuN-positive fraction and a larger BrdU-only fraction in the CaEDTA-treated FUS group, whereas the GFAP-positive fraction was broadly similar across groups. Together, these findings indicate that extracellular zinc chelation attenuated FUS-associated neurogenic responses in the DG, whereas the ZnEDTA control did not reproduce this effect, supporting a contribution of labile zinc availability to the full magnitude of the FUS-associated dentate neurogenic response.

### Zinc chelation suppresses FUS-associated increases in BDNF, ZIP-3, and Piezo1 protein expression

**Figure 5:**
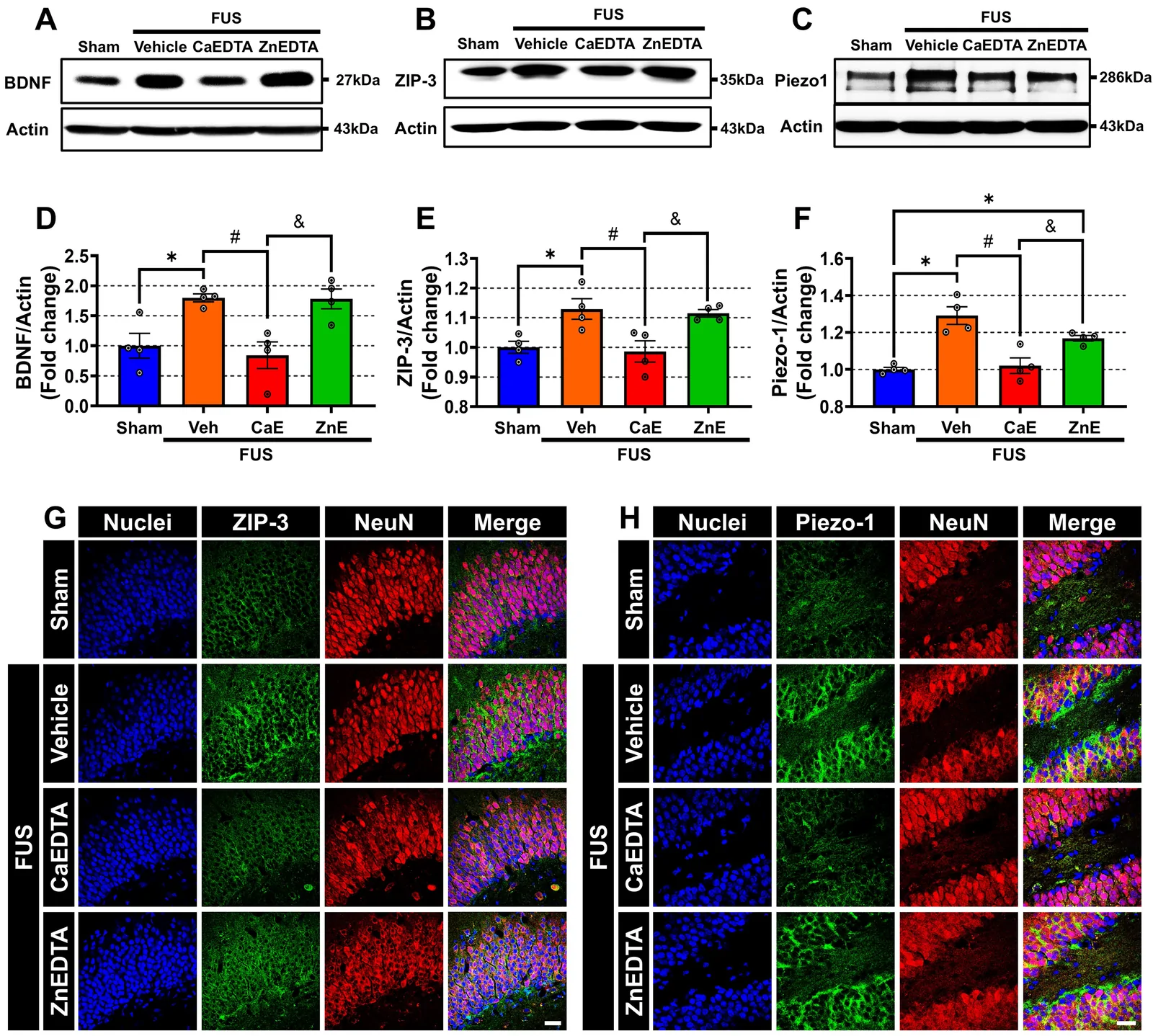
** BDNF, ZIP-3, and Piezo1 protein analysis in the hippocampus after FUS with or without zinc chelation. (A–C)** Representative western blots of BDNF **(A)**, ZIP-3 **(B)**, Piezo1 **(C)**, and β-actin in hippocampal tissue collected 24 h after FUS from the sham-operated, vehicle-treated FUS, CaEDTA-treated FUS, and ZnEDTA-treated FUS groups. **(D–F)** Densitometric quantification of BDNF **(D)**, ZIP-3 **(E)**, and Piezo1 **(F)**, normalized to β-actin. Data are presented as mean ± SEM (n = 4 rats per group). Panels D–F were analyzed using one-way ANOVA followed by post hoc multiple-comparison tests as described in the Methods. *P < 0.05 versus the sham-operated group; #P < 0.05 versus the vehicle-treated FUS group; &P < 0.05 versus the CaEDTA-treated FUS group. **(G, H)** Representative immunofluorescence images of the DG collected 24 h after FUS from the vehicle-treated FUS and CaEDTA-treated FUS groups, stained for ZIP-3 (green) and NeuN (red) **(G)**, or Piezo1 (green) and NeuN (red) **(H)**. Merged images show partial overlap of ZIP-3 or Piezo1 signals with NeuN-positive cells. Scale bar = 20 μm.

To examine candidate molecular changes associated with the zinc-sensitive hippocampal response to FUS, we next measured BDNF, ZIP-3, and Piezo1 protein expression in hippocampal tissue collected 24 h after FUS. BDNF is a well-established promoter of neural progenitor survival, differen-tiation, and synaptic plasticity. ZIP-3 is a zinc transporter that contributes to cellular zinc uptake and homeostasis, whereas Piezo1 has recently emerged as a transducer of mechanical signals into intracellular biochemical responses, making it a compelling candidate for mediating FUS-driven effects. Western blot analysis showed that the vehicle-treated FUS group had higher BDNF, ZIP-3, and Piezo1 protein levels than the sham-operated group (Figure 5A-F). In contrast, these FUS-associated increases were attenuated in the CaEDTA-treated FUS group, whereas the ZnEDTA-treated FUS group showed protein levels comparable to those of the vehicle-treated FUS group. Densitometric analysis confirmed significant increases in BDNF, ZIP-3, and Piezo1 in the vehicle-treated FUS group and significant reductions in the CaEDTA-treated FUS group relative to the vehicle-treated FUS group (Figure 5D-F). Representative immunofluorescence images obtained from the DG 24 h after FUS showed stronger ZIP-3 and Piezo1 signals in the vehicle-treated FUS group, with partial overlap with NeuN-positive cells, whereas these signals appeared weaker in the CaEDTA-treated FUS group. The ZnEDTA-treated FUS group showed a staining pattern similar to that in the vehicle-treated FUS group (Figure 5G-H). Together, these findings indicate that unilateral FUS was associated with zinc-sensitive increases in hippocampal BDNF, ZIP-3, and Piezo1 protein expression, identifying these molecules as candidate components of the broader FUS-associated tissue response.

### ZnT3-associated vesicular zinc contributes to the early neurogenic response to unilateral FUS

**Figure 6:**
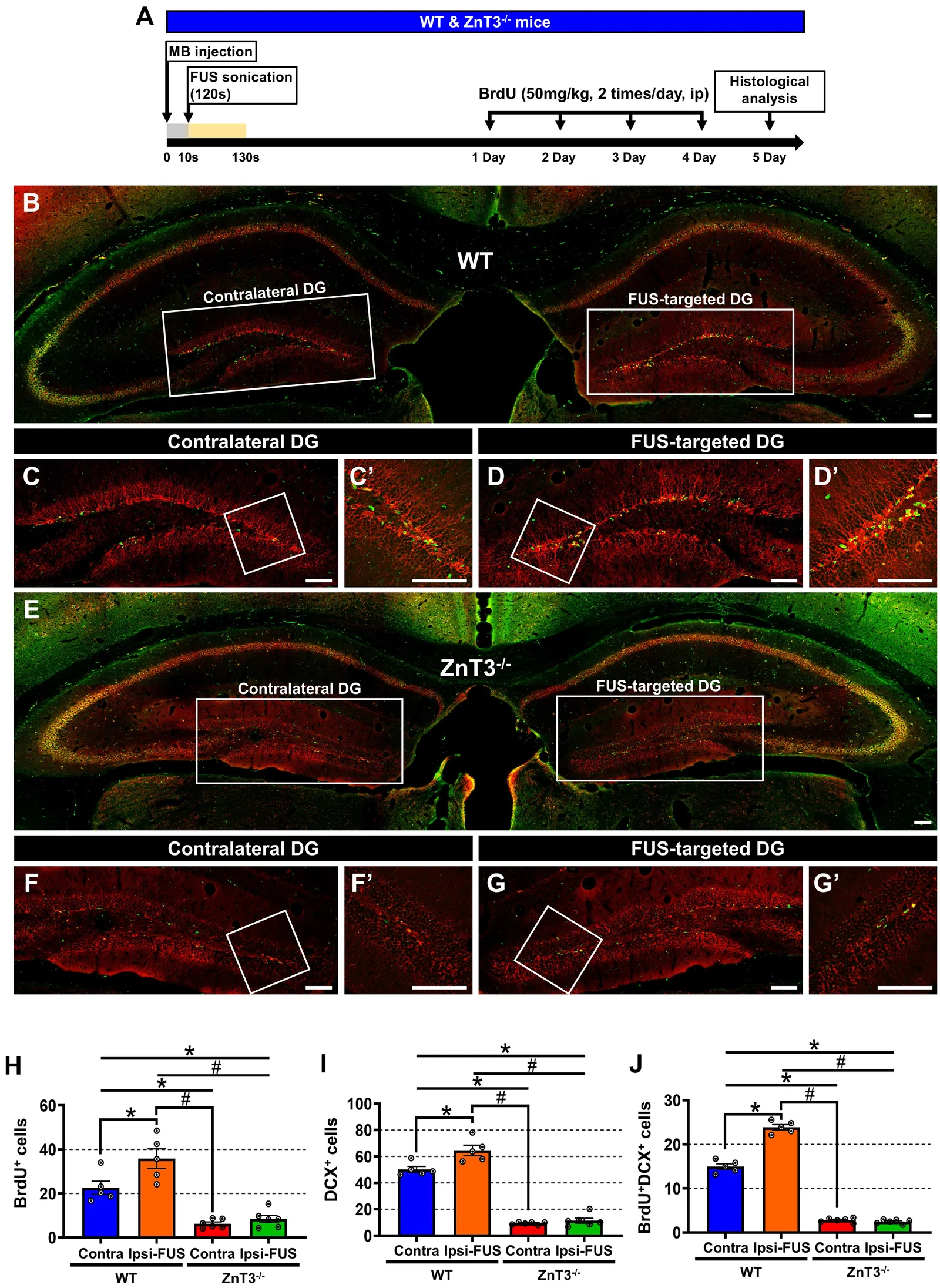
** BrdU and DCX labeling in WT and** ZnT3^˗/˗^** mice after unilateral FUS. (A)** Schematic of the experimental timeline. **(B–G)** Representative coronal sections of the DG from WT** (B–D)** and ZnT3^˗/˗^** (E–G)** mice stained for BrdU (green) and DCX (red) on day 5 after FUS.** (B, E)** Low-magnification images showing the matched contralateral and FUS-targeted ipsilateral DG within the same animals. Scale bars = 200 μm. White boxes indicate the SGZ/GCL region shown at higher magnification in **(C, D)** for WT mice and** (F, G)** for ZnT3^˗/˗^ mice.** (C, F)** Matched contralateral DG. **(D, G)** FUS-targeted ipsilateral DG. Scale bars = 50 μm.** (C′, D′, F′, G′)** Higher-magnification insets of the corresponding boxed regions showing representative BrdU/DCX double-positive cells. Scale bars = 20 μm.** (H–J)** Quantification of BrdU-positive cells** (H)**, DCX-positive cells **(I)**, and BrdU/DCX double-positive cells **(J)** in the matched contralateral and FUS-targeted ipsilateral DG of WT and ZnT3^˗/˗^ mice on day 5 after FUS. Data are presented as mean ± SEM (n = 5 for WT, n = 6 for ZnT3^˗/˗^ mice). Hemisphere and genotype effects were analyzed using two-way mixed ANOVA followed by Bonferroni-corrected post hoc comparisons. *P < 0.05 versus the matched contralateral DG within the same genotype; ^#^P < 0.05 versus the FUS-targeted ipsilateral DG of WT mice.

Given that extracellular zinc chelation attenuated the FUS-associated neurogenic response, we next examined whether ZnT3-associated vesicular zinc contributes to this effect using ZnT3^˗/˗^ mice, which lack the zinc transporter responsible for packaging zinc into synaptic vesicles at glutamatergic terminals. As shown in Figure 6A, adult ZnT3^˗/˗^ mice and their WT littermates underwent unilateral FUS targeting of the hippocampus. All animals received BrdU injections after sonication to label dividing cells (Figure 6A). Representative images obtained 5 days later showed greater BrdU and DCX labeling in the FUS-targeted ipsilateral DG than in the matched contralateral DG in WT mice, whereas this hemispheric difference was not evident in ZnT3^˗/˗^ mice (Figure 6B-G). Quantitative analysis confirmed significant increases in BrdU-positive cells, DCX-positive cells, and BrdU/DCX double-positive cells in the FUS-targeted ipsilateral DG of WT mice. In contrast, none of these measures showed a significant hemispheric increase in ZnT3^˗/˗^ mice. Moreover, the numbers of BrdU-positive cells, DCX-positive cells, and BrdU/DCX double-positive cells in the FUS-targeted ipsilateral DG were significantly lower in ZnT3^˗/˗^ mice than in WT mice (Figure 6H-J). Together, these findings show that the increase in proliferative and immature neuronal markers observed in response to unilateral FUS in WT mice was not reproduced in ZnT3^˗/˗^ mice, consistent with an important contribution of ZnT3-associated vesicular zinc to the early neurogenic response to FUS.

### Exploratory hippocampal transcriptomic profiles differ by FUS exposure and ZnT3 genotype

**Figure 7:**
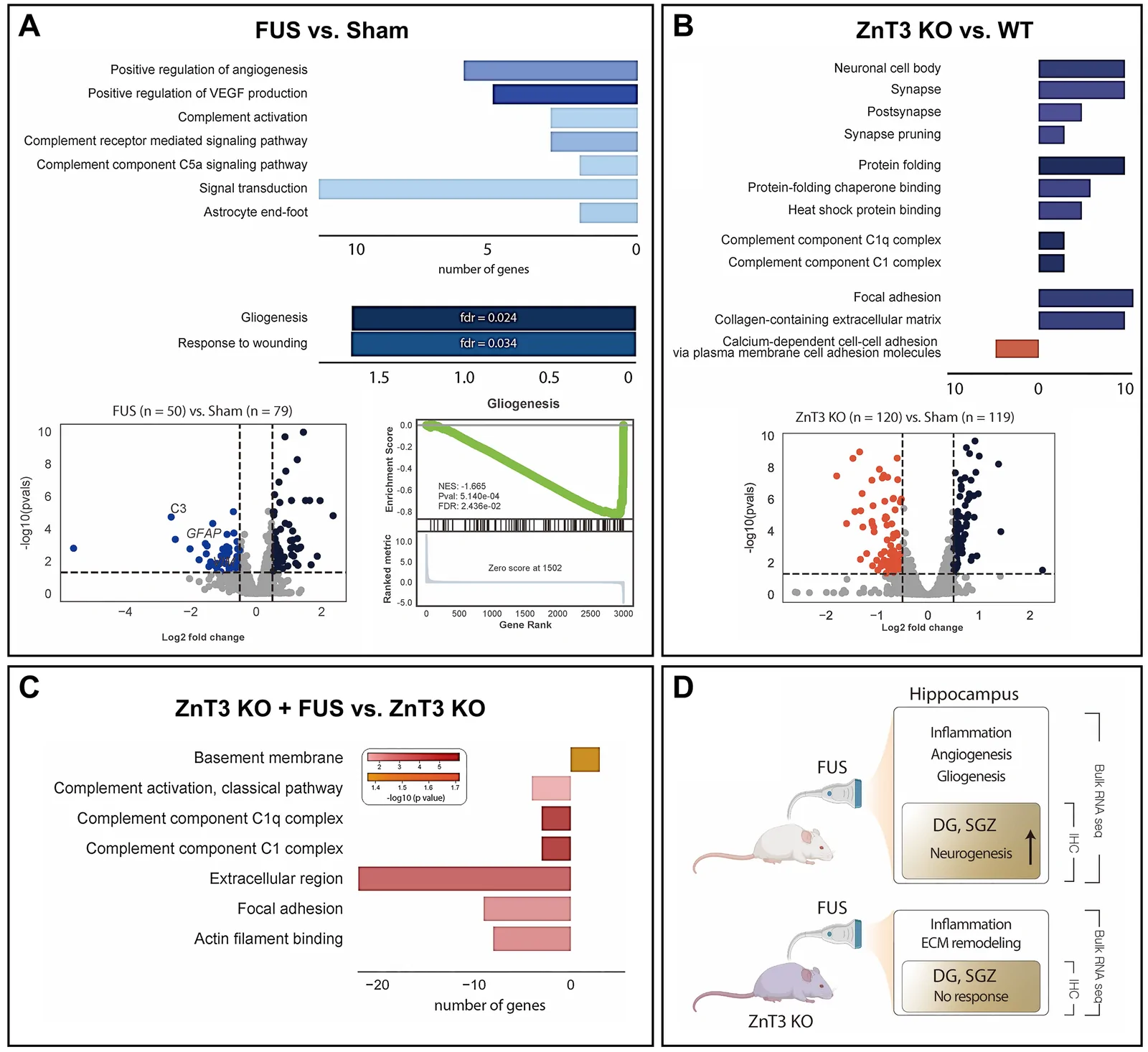
** Exploratory bulk RNA-seq analysis of hippocampal tissue according to FUS treatment and ZnT3 genotype. (A)** Gene Ontology enrichment analysis and volcano plot for the WT-FUS versus WT-sham comparison, showing selected differentially expressed genes and enriched terms related to angiogenesis, gliogenesis, and neuroglial cell differentiation. **(B)** Gene Ontology enrichment analysis and volcano plot for the ZnT3^˗/˗^-sham versus WT-sham comparison, showing selected enriched terms related to complement activation, synapse pruning, and calcium signaling. **(C)** Gene Ontology enrichment analysis for the ZnT3^˗/˗^-FUS versus ZnT3^˗/˗^-sham comparison, showing selected enriched terms related to inflammatory responses and extracellular matrix organization. **(D)** Schematic summary of the exploratory transcriptomic comparisons shown in **(A–C)**.

To further examine whether hippocampal tissue responses to FUS differed between ZnT3 genotypes, we performed exploratory bulk RNA-seq analysis followed by Gene Ontology enrichment analysis. At the transcript level, Piezo1 and BDNF exhibited upward numerical trends in WT-FUS samples, although these changes did not reach significance in the bulk RNA-seq dataset. In the WT-FUS versus WT-sham comparison, 50 genes were upregulated and 79 genes were downregulated, and enriched terms included positive regulation of angiogenesis, positive regulation of VEGF production, complement-related signaling, signal transduction, astrocyte end-foot, gliogenesis, and response to wounding (Figure 7A). These findings suggest that FUS in the WT hippocampus was associated with transcriptional programs related to vascular and glial remodeling. In the ZnT3^˗/˗^ -sham versus WT-sham comparison, the differentially enriched terms were primarily related to synaptic compartments, complement-associated pathways, protein folding/chaperone activity, and extracellular matrix or cell-adhesion processes (Figure 7B), indicating that ZnT3 deletion was associated with a distinct baseline hippocampal transcriptional profile. In the ZnT3^˗/˗^-FUS versus ZnT3^˗/˗^-sham comparison, the enriched terms predominantly reflected complement activation, extracellular region, focal adhesion, actin filament binding, and basement membrane (Figure 7C). Notably, the angiogenic and gliogenic signatures highlighted in the WT-FUS comparison were not prominent in the ZnT3^˗/˗^-FUS comparison. Taken together, although exploratory and descriptive, these bulk transcriptomic data suggest that FUS in WT mice was associated with angiogenic, complement-related, astrocyte-associated, and neuroglial remodeling signatures, whereas the response in ZnT3^˗/˗^ mice shifted toward complement/inflammatory and extracellular matrix-related programs, consistent with the genotype-dependent histological differences observed in Figure 6 and summarized schematically in Figure 7D.

## Discussion

In this study, transient FUS combined with microbubbles produced localized BBB modulation in the hippocampus and was associated with increased zinc-related signals, including TSQ-detectable fluorescence and ZnT3 immunoreactivity, together with enhanced dentate gyrus neurogenesis. Both pharmacological chelation of extracellular zinc and genetic loss of ZnT3 attenuated these FUS-associated neurogenic effects. Taken together, these findings support the interpretation that intact zinc availability contributes to the full expression of the hippocampal neurogenic response associated with FUS-mediated BBB modulation.

Our findings extend earlier reports showing that transcranial FUS with microbubbles can stimulate adult hippocampal neurogenesis. Scarcelli et al. 23 first showed that FUS-mediated BBB modulation enhances cell proliferation and newborn neuron labeling in the adult mouse hippocampus. Subsequent studies further demonstrated that this neurogenic effect depends on FUS-mediated BBB modulation and that FUS can improve hippocampal neurogenesis and cognition in disease models, including in experimental dementia paradigms 24,25. More recent work has also shown that low-intensity FUS-mediated BBB modulation can activate endogenous neural stem cell responses and be coupled to the delivery of biologics that influence hippocampal plasticity and repair 26-29. Consistent with this literature, we observed increased BrdU and DCX labeling after unilateral FUS. Together with BrdU⁺/NeuN⁺ newborn-neuron labeling, these histological endpoints support enhanced adult hippocampal neurogenesis, but they do not establish whether the newborn neurons functionally integrate into hippocampal circuits or contribute to measurable cognitive outcomes. Direct functional and hippocampus-dependent behavioral assessments will therefore be required to determine the physiological relevance of this FUS-associated neurogenic response. Within this interpretive scope, the main contribution of the present study is that it identifies zinc availability as an important determinant of whether this pro-neurogenic response can occur.

This interpretation is consistent with a substantial body of literature implicating zinc in adult hippocampal neurogenesis. Reduced zinc availability impairs progenitor proliferation, neuroblast formation, and neuronal differentiation, whereas zinc supplementation can enhance these processes 35-39,45-48. ZnT3, which is required for vesicular zinc loading at glutamatergic terminals, has also been shown to modulate proliferation and neuronal differentiation in the adult hippocampus 35,36. Moreover, environmental enrichment fails to fully enhance hippocampal neurogenesis in ZnT3-deficient mice, supporting the idea that vesicular zinc signaling contributes to activity- or experience-dependent recruitment of the dentate neurogenic niche 49. Viewed in this context, the present pharmacological and genetic data fit well with the broader concept that adequate zinc availability is necessary for adult hippocampal neurogenic competence.

At the same time, our data should not be interpreted as proving that zinc is a uniquely FUS-specific instructive signal. An alternative, non-mutually exclusive interpretation is that zinc acts as a permissive regulator of dentate neurogenic competence. Basal adult hippocampal neurogenesis is sensitive to zinc status, and ZnT3-dependent vesicular zinc signaling contributes not only to baseline proliferation and neuronal differentiation but also to enrichment-associated neurogenic enhancement 36,37,45,49. Within this framework, the inability of CaEDTA-treated or ZnT3-deficient animals to show a robust FUS-associated increase in neurogenic markers may reflect a reduced baseline niche responsiveness, in addition to any stimulus-coupled role of zinc during or after FUS exposure. Accordingly, the present data support an important contribution of zinc availability and ZnT3 status, but they do not fully distinguish permissive from instructive roles of zinc in the FUS-responsive dentate niche.

Importantly, the zinc-dependent contribution identified here should be interpreted within the broader biological context of FUS-mediated BBB modulation. In addition to altered zinc availability, FUS-mediated BBB modulation can involve transient endothelial barrier changes, vascular permeability responses, sterile inflammatory signaling, complement activation, glial responses, and extracellular matrix remodeling 19,21,22,50,51. These inflammatory, vascular, and glial responses should not be interpreted as being necessarily downstream of zinc signaling; rather, they may represent parallel or complementary responses to FUS-mediated BBB modulation that also shape the adult hippocampal niche. Thus, non-zinc FUS-mediated BBB modulation mechanisms may contribute to FUS-associated neurogenic remodeling independently of, or in concert with, the zinc-dependent mechanism proposed in the present study.

**Figure 8:**
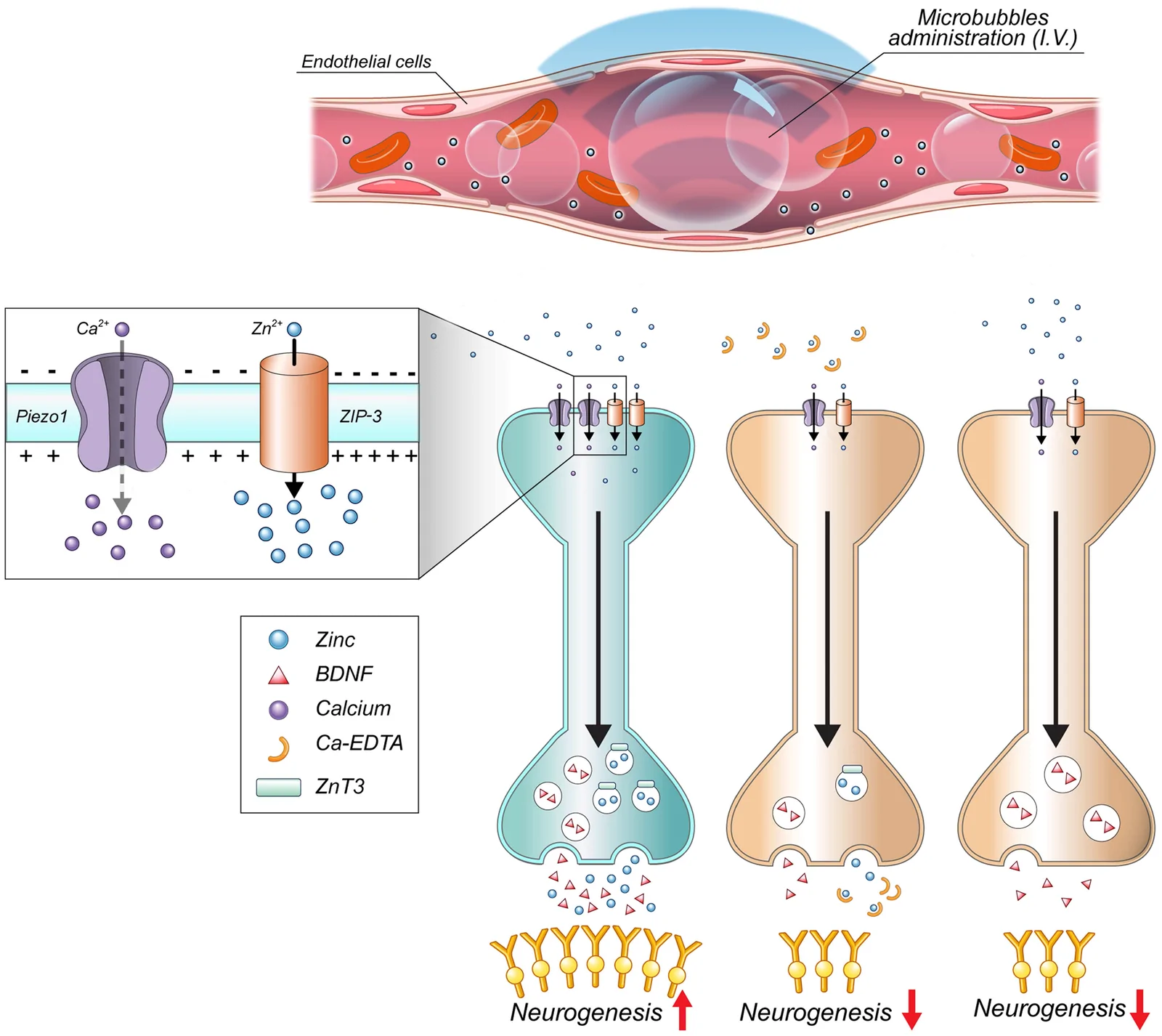
** Proposed model linking FUS-induced BBB modulation, zinc-associated signaling, and adult hippocampal neurogenesis.** Schematic illustration integrating the main findings of the present study. The upper panel depicts FUS with intravenously administered microbubbles at the cerebral microvasculature. The inset illustrates proposed membrane-associated zinc and calcium signaling involving ZIP-3 and Piezo1. The lower schematics summarize three experimental contexts: intact ZnT3-associated vesicular zinc signaling (left), extracellular zinc chelation with CaEDTA (middle), and loss of ZnT3-associated vesicular zinc signaling (right). Upward and downward arrows indicate the relative neurogenesis outcomes observed across conditions. Experimentally supported findings incorporated into this model include FUS-associated increases in BBB permeability, TSQ-detectable zinc fluorescence, ZnT3 immunoreactivity, and BDNF, ZIP-3, and Piezo1 protein levels, as well as attenuation of neurogenesis by CaEDTA treatment and in ZnT3^˗/˗^ mice. The transvascular entry of zinc or BDNF and the specific channel-mediated Zn²⁺ or Ca²⁺ fluxes shown in the schematic are proposed mechanisms and were not directly measured in the present study.

Within this broader framework, several complementary pathways may link FUS-associated BBB modulation to zinc-dependent neurogenic remodeling (Figure 8). FUS with microbubbles is known to transiently increase BBB permeability and can alter endothelial transport, vascular permeability, and local tissue responses when applied within an appropriate acoustic window 8-14,19-22. Because most circulating zinc is protein-bound, the extent to which blood-derived zinc directly enters the hippocampal parenchyma after FUS remains unresolved. Nevertheless, even relatively small changes in labile Zn²⁺ can affect neural signaling because zinc functions as a neuromodulator in the central nervous system 30-32. In parallel, the present study showed increased TSQ fluorescence and increased ZnT3 immunoreactivity after FUS, consistent with altered vesicular zinc-associated signaling in hippocampal terminals. Zinc has also been reported to transactivate TrkB-related signaling and to modulate mossy fiber synaptic physiology, while BDNF is a well-established regulator of progenitor survival, neuronal differentiation, and maturation 34,52-55. Together, these observations support a working model in which FUS-associated BBB modulation and hippocampal zinc-associated responses converge to favor a neurogenesis-permissive niche state, although the source, route, and temporal ordering of zinc signals were not directly resolved in the present study.

Among the candidate molecular responses examined here, Piezo1 is of particular interest because it is a mechanically activated, Ca²⁺-permeable cation channel 56,57. Piezo1 protein expression increased after FUS, and this increase was attenuated by extracellular zinc chelation, consistent with its interpretation as a zinc-sensitive molecular correlate of the FUS response. Prior studies have shown that Zn²⁺ can modulate Piezo1 biophysical properties, specifically slowing inactivation under defined electrophysiological conditions 56, which may prolong channel activity during mechanical stimulation. Accordingly, increased zinc availability following FUS may potentiate Piezo1-mediated Ca²⁺ entry and extend intracellular Ca²⁺ transients. Prolonged Ca²⁺ signaling could, in turn, engage activity-dependent transcriptional programs through factors such as CREB and immediate early genes (e.g., *EGR1*), which were upregulated in a zinc-dependent manner in our study. However, the present study assessed only Piezo1 protein expression and did not directly assess Piezo1 channel function using electrophysiological measurements, mechanically evoked current recordings, inactivation-kinetic analyses, or Ca²⁺ imaging. Therefore, our findings support an association between FUS, zinc availability, and increased Piezo1 expression but not direct evidence that zinc functionally regulates Piezo1 activity *in vivo* under the present conditions. This distinction is particularly important because astrocytic Piezo1-mediated mechanotransduction has been implicated in adult neurogenesis and cognitive regulation 58. Whether neuronal Piezo1, astrocytic Piezo1, or both contribute to the present phenotype will require cell-type-specific and functional studies.

BDNF represents another plausible component of the zinc-associated response observed after FUS. In the adult hippocampus, BDNF supports basal neurogenesis and promotes the survival, differentiation, and maturation of adult-born neurons 52-54. Zinc has also been linked to activity-dependent neurotrophic signaling, including TrkB-related pathways 34,55. In the present study, BDNF protein expression increased after FUS, and this increase was attenuated by CaEDTA treatment, consistent with the possibility that zinc contributes to a trophic niche state after FUS. Nevertheless, because we did not directly test the necessity of BDNF signaling in this model, the present data should be interpreted as identifying BDNF as a candidate zinc-sensitive molecular response rather than establishing a validated linear zinc–BDNF mechanism.

The exploratory bulk RNA-seq analysis further suggested that hippocampal tissue responses to FUS differed between ZnT3 genotypes. In the WT hippocampus, FUS was associated with enriched terms related to angiogenesis, complement-associated signaling, astrocyte-associated processes, gliogenesis, and response to wounding, whereas in the ZnT3^˗/˗^ hippocampus, the FUS-associated profile was dominated by complement activation, extracellular matrix-related terms, basement membrane, focal adhesion, and actin filament binding. These patterns are broadly consistent with prior reports showing that microbubble-enhanced FUS-mediated BBB modulation can evoke transient sterile inflammation, glial activation, endothelial or vascular remodeling, and angiogenic responses without overt chronic tissue damage when delivered within safe acoustic parameters 19-22,50,51,59. They are also compatible with the broader concept that angiogenesis and neurogenesis are coupled processes within the adult neurogenic niche 60,61. However, the present transcriptomic findings are exploratory and descriptive, and they do not independently establish causality between any single enriched pathway and the histological neurogenesis phenotype observed in this study.

From a translational perspective, the present findings raise the possibility that zinc status may influence the biological response to FUS-based BBB modulation. FUS-mediated BBB modulation is increasingly being investigated as a therapeutic platform for neurological disease, particularly Alzheimer’s disease, where it has been used to enable regional BBB modulation and to enhance the delivery of therapeutic agents 15-18. Because zinc homeostasis has been implicated in central nervous system disease, including Alzheimer’s disease 31,33, inter-individual differences in zinc availability could plausibly modify regenerative or neuroplastic responses to FUS. Although this possibility remains speculative, it suggests that zinc-responsive pathways may merit consideration in future studies designed to optimize FUS-based regenerative or drug-delivery strategies.

Some limitations should be emphasized. First, the source and mode of zinc signaling remain unresolved. CaEDTA primarily targets extracellular/labile zinc and can reduce neuronal zinc availability through extracellular chelation, whereas ZnT3 deficiency reflects lifelong disruption of vesicular zinc-associated physiology and may include developmental or compensatory effects. Thus, the present experiments do not distinguish synaptically released vesicular zinc from a possible contribution of blood-derived zinc after FUS-mediated BBB modulation. Second, the molecular data identify candidate correlates rather than a validated sequence of causal events; the present study did not test the necessity of BDNF, ZIP-3, or Piezo1, nor did it directly assess Piezo1 function or downstream Ca²⁺-dependent signaling. Third, the contralateral hippocampus served as a matched intra-animal comparator rather than a fully independent negative control, and the ZnT3 histological and transcriptomic experiments were conducted in separate cohorts. Fourth, the size of the RNA-seq dataset was modest, and the transcriptomic analyses were exploratory. Finally, only male animals were examined, and the study did not assess behavioral outcomes or direct functional integration of newborn neurons. Collectively, these limitations constrain causal inference, functional interpretation, and generalizability and should be addressed in future studies.

Overall, the present findings support a model in which transient FUS-mediated BBB modulation engages a zinc-sensitive hippocampal tissue response that is permissive for enhanced adult hippocampal neurogenesis. Within this model, zinc should be viewed not as a definitively established single upstream trigger but as an important molecular determinant of whether the dentate niche can mount the neurogenic response associated with FUS exposure.

## Conclusion

The present study indicates that the early neurogenic response associated with unilateral FUS in the adult hippocampus is influenced by intact zinc availability and is markedly attenuated by extracellular zinc chelation or ZnT3 deficiency. We propose a cautious conceptual framework in which FUS-mediated BBB modulation is accompanied by alterations in hippocampal zinc-associated signaling and by increased BDNF, ZIP-3, and Piezo1 protein expression, together supporting a neurogenesis-permissive dentate niche state rather than establishing a single verified signaling cascade. These findings broaden the biological significance of FUS-mediated BBB modulation beyond targeted delivery alone and suggest that zinc-responsive pathways may influence how the adult hippocampus responds to acoustic BBB modulation.

## Data Availability

The data that support the findings of this study are available from the corresponding author upon reasonable request.

## Abbreviations

FUS: focused ultrasound
BBB: blood–brain barrier
BDNF: brain-derived neurotrophic factor
ZIP-3: Zrt-/Irt-like protein 3
KO: knockout
CaEDTA: calcium-saturated EDTA
ZnEDTA: zinc-saturated EDTA
MRI: magnetic resonance imaging
DG: dentate gyrus
PBS: phosphate-buffered saline
PFA: paraformaldehyde
WT: wild type
BrdU: bromodeoxyuridine
NeuN: neuronal nuclei
GFAP: glial fibrillary acidic protein
DAPI: 4′,6-diamidino-2-phenylindole
DCX: doublecortin
TSQ: N-(6-methoxy-8-quinolyl)-para-toluenesulfonamide
DAB: diaminobenzidine
FC: fold change
FDR: false discovery rate
SGZ: subgranular zone
ZnT3: zinc transporter 3
